# Allele-Specific PCR for PIK3CA Mutation Detection Using Phosphoryl Guanidine Modified Primers

**DOI:** 10.3390/diagnostics13020250

**Published:** 2023-01-09

**Authors:** Alexey S. Chubarov, Igor P. Oscorbin, Lidiya M. Novikova, Maxim L. Filipenko, Alexander A. Lomzov, Dmitrii V. Pyshnyi

**Affiliations:** Institute of Chemical Biology and Fundamental Medicine, SB RAS, 8 Lavrentiev Avenue, Novosibirsk 630090, Russia

**Keywords:** mutation detection, PIK3CA mutations, allele-specific PCR, modified oligonucleotides, phosphoryl guanidine oligonucleotide

## Abstract

Phosphoryl guanidine (PG) is the novel uncharged modification of internucleotide phosphates of oligonucleotides. Incorporating PG modification into PCR primers leads to increased discrimination between wild-type and mutated DNA, providing extraordinary detection limits in an allele-specific real-time polymerase chain reaction (AS-PCR). Herein, we used PG-modification to improve the specificity of AS primers with unfavorable Pyr/Pur primer’s 3′-end mismatch in the template/primer complex. Two mutations of the PIK3CA gene (E542K, E545K) were chosen to validate the advantages of the PG modification. Several primers with PG modifications were synthesized for each mutation and assessed using AS-PCR with the plasmid controls and DNA obtained from formalin-fixed paraffin-embedded (FFPE) tissues. The assay allows the detection of 0.5% of mutated DNA on the wild-type DNA plasmid template’s background with good specificity. Compared with ddPCR, the primers with PG-modification demonstrated 100% specificity and 100% sensitivity on the DNA from FFPE with mutation presence higher than 0.5%. Our results indicate the high potential of PG-modified primers for point mutation detection. The main principle of the developed methodology can be used to improve the specificity of primers regardless of sequences.

## 1. Introduction

Somatic mutation detection is a necessary procedure before the appointment for targeted anti-cancer therapy. *PIK3CA*, the gene encodes the alpha catalytic subunit of phosphatidylinositol-4,5-bisphosphate 3-kinase (PI3K), acting in signaling pathways responsible for cell metabolism, proliferation, viability, and adhesion. *PIK3CA* mutations are observed frequently in a broad spectrum of cancers, including the most common malignancies worldwide in breast, endometrial, and colorectal tumors [1,2,3,4]. Point mutations in *PIK3CA* are mostly clustered in exon 9 (codons 542, 545) and exon 20 (codon 1047), encoding the helical and kinase domains of the PI3K p110α protein, respectively [5,6]. *PIK3CA* is altered in 36% of breast carcinoma and 14% of solid malignant tumors in general [4]. The *PIK3CA* E542K and E545K mutations were found in 1.7% and 2.9% of solid malignancies [4]. Various methodologies were used for *PIK3CA* mutation detection as quantitative polymerase chain reaction (qPCR), droplet digital PCR (ddPCR), allele-specific PCR (AS-PCR), melting curve analysis, next-generation sequencing (NGS), etc. [7,8,9,10,11,12,13,14,15,16,17,18,19,20,21,22,23,24]. NGS is one of the most powerful methods that allow the simultaneous detection of multiple mutations, including unknown alterations [25,26,27]. Several commercial NGS panels for somatic mutation testing are available on the market. However, NGS is time-consuming, costly, and requires highly-trained personnel, even in the case of relatively simple amplicon sequencing, while general clinical practice prefers rapid, cheap, and simple methods. Quantitative PCR allows to perform testing in conditions with limited resources, and this method is also commonly used for other purposes, such as testing of inflectional diseases. Therefore, NGS and qPCR occupy different niches and can coexist as mutually supportive methods, while each hospital can choose the most approach in accordance with local demands. Digital PCR is a relatively modern method with superior sensitivity but a higher cost per single analysis. Thus, ddPCR instruments and reagent kits are much more expensive, which only large, well-funded hospitals can afford. One of the disadvantages, which can yet be overcome, is the generation of false positive results that could appear even testing no-template controls [28]. Taking that in mind, the usage of digital PCR also requires well-trained personnel. For these criteria, AS-PCR fits well, considering its lasting usage in clinics and sufficient analytical sensitivity (~1% of variant allele frequency, VAF, is detectable). Nowadays, most general hospitals are equipped with thermal cyclers, making it easy to perform PCR analysis.

**Figure 1:**
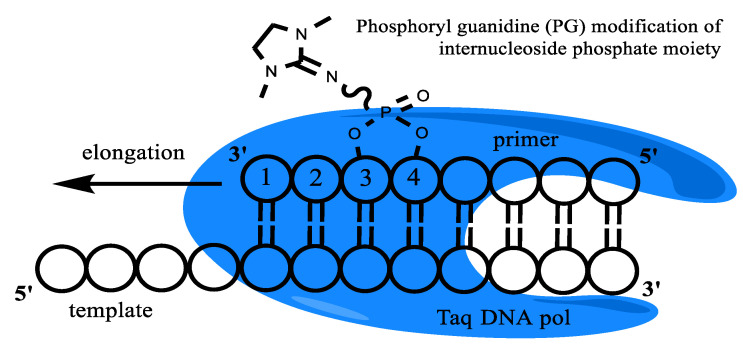
Taq-polymerase and template/modified primer complex schematic representation. An example of PG modification is presented.

In AS-PCR, DNA molecules are selectively amplified using a unique AS primer, which provides discrimination between mutant and wild-type DNA. However, the AS primer’s design is a featured area complicated by low analytical specificity. For this reason, several nucleotide substitutions can be added at the 3′-terminus of the primer to increase the specificity of mutated DNA detection [29,30,31,32]. The other options are to use partly or fully modified oligonucleotides as an AS-PCR primer. For instance, locked nucleic acids (LNA), phosphorothioate, non-natural bases, methyl phosphotriester, and peptide nucleic acids (PNA) are currently used for qPCR [32,33,34,35,36,37,38,39,40,41,42]. Recently, advanced phosphate-modified oligonucleotides, namely phosphoryl guanidine oligonucleotides (PGOs), have been synthesized and used for AS-PCR [43,44,45]. Such uncharged-phosphate modification can be easily introduced into various primers′ positions by standard machine phosphoramidite oligonucleotide synthesis. PG-modification (1,3-dimethylimidazolidine-2-imine group, Figure 1) does not perturb the DNA structure [46,47] and can be used for various applications [45,48,49,50,51,52,53].

In our previous work [45], we successfully used PG-modified primers in AS-PCR for *KRAS* gene mutation detection. We presented the first results of a new oligonucleotide class investigation (Phosphoryl guanidine) for AS-PCR. We have shown that phosphoryl guanidine modification increases the specificity of mutation detection. However, insufficient specificity was obtained for G12V KRAS mutation detection. The AS primers for G12V KRAS have unfavorable 3′-end mismatch (Pyr/Pur) in the template/primer complex, which leads to low WT and mutant DNA discrimination efficiency. Moreover, the experiments were done only on the plasmid model system, while the actual DNA for somatic mutation testing was purified from formalin-fixed tissues (FFPE). Such DNA is highly chemically modified with numerous cross-links and lesions, which leads to reduced amplification efficiency. Therefore, PCR results obtained on a plasmid model cannot be directly extrapolated to real clinical samples. The PG-modified primers need to be validated on FFPE-obtained DNA.

To address these challenges, in this, only the second paper on PGO investigation for AS-PCR application, we applied the same strategy and PGO in AS-PCR to improve the low-efficient AS primers with Pyr/Pur 3′-end mismatch in template/primer complex. As a model, we have chosen E542K and E545K mutations in the *PIK3CA* gene and designed AS primers for qPCR with TaqMan probes. The influence of the PG modification on the PCR efficacy, specificity, and sensitivity was studied using plasmid templates. The performance of the assay was tested not only on the model plasmid system but in DNA samples from FFPE. The diagnostic sensitivity, specificity, and repeatability of the assay were also assessed. The successful demonstration of high specific mutation detection is reported. We believe that the present study shows the great potential of PG modification and would be interesting for all researchers applying AS-PCR and other technologies for somatic mutation detection.

## 2. Materials and Methods

### 2.1. Synthesis and Isolation of Oligonucleotides

Oligonucleotides were synthesized in an ASM-800 automated synthesizer (Biosset, Novosibirsk, Russia) according to the standard protocol of the 2-cyanoethyl phosphoramidite method. Oligonucleotides containing phosphoryl guanidine modification were synthesized using the protocol described previously by LLC NooGen [43,44]. The purification of oligonucleotides by reverse phase high-pressure liquid chromatography (RP-HPLC) was performed on the Agilent 1200 series chromatograph (Agilent, Santa Clara, CA, USA) on a column (4.6 × 150 mm) containing the Eclipse XDB-C18 sorbent (5 μm) (Agilent, Santa Clara, CA, USA) with a 0–90% linear gradient of acetonitrile concentration in 0.02 M triethylammonium acetate solution for 30 min at a flow rate of 1.5 mL/min. Fractions containing the target product were evaporated in vacuo. Coevaporation with ethanol removed the bulk of triethylammonium acetate. To remove the protecting dimethoxytrityl group, the purified oligonucleotides were treated with 80% acetic acid (25 °C, 7 min). Then oligonucleotides were concentrated, followed by precipitation with 2% LiClO_4_ in acetone, washing with pure acetone, and desiccation under a vacuum. After desiccation, the oligonucleotides were dissolved in 0.1 mL of deionized water and stored at –20 °C. The concentration of oligonucleotides in solutions was determined on a UV-1800 spectrometer (Shimadzu, Kyoto, Japan) according to the procedure described earlier [54].

### 2.2. Plasmid Standards

The control plasmids contained a partial sequence of the wild-type *PIK3CA* gene (GenBank ID NG_012113.2) or *PIK3CA* gene with mutations in exon 9, e.g., E545K c.1633G>A (COSMIC ID COSM763) and E542K c.1624G>A (COSMIC ID COSM760), were used to assess method sensitivity and were constructed by Shanghai RealGene Biotech, Inc (Shanghai, China). The plasmid backbone was pBlueScript SK (+). All control plasmids were sequenced before the usage, and the inserts of the PIK3CA gene were identical to the theoretical sequences. Before use, all control plasmids were purified, linearized by digestion with BamHI restriction endonuclease, and quantified using NanoDrop Lite A4 spectrophotometer (Thermo Fisher Scientific, Waltham, MA, USA) and droplet digital PCR using primers and probe for the beta-lactamase gene: Lac-U 5′-CGTCGTTTGGTATGGCTTCATTC-3′, Lac-R 5′-AGGACCGAAGGAGCTAACCG-3′, Lac-P 5′-HEX-CGGTTCCCAACGATCAAGGCGAG-BHQ2-3′.

### 2.3. qPCR

Reactions were performed in 20 µL containing 1× PCR-buffer (65 mM Tris–HCl, pH 8.9, 24 mM (NH_4_)_2_SO_4_, 0.05 % Tween-20, 3 mM MgSO_4_), 0.2 mM dNTP, 600 nM primers, 100 nM fluorescent hydrolysis probe (TaqMan), DNA template, and 1 U of Taq-polymerase (Biosan, Novosibirsk, Russia). Control plasmids were used as DNA templates at the concentration indicated below. Amplification was carried out in the CFX96 Real-Time PCR Detection System (Bio-Rad, Hercules, CA, USA) according to the following program: 95 °C for 3 min followed by 45 cycles of 95 °C for 10 s, and 60 °C for 40 s with a collection of fluorescent signals at the FAM channel. Reactions were carried out in triplicates and performed several times on separate occasions. Average quantification cycle (Cq) ± standard deviation (SD) values are given in the tables. PCR analysis was performed using a forward primer 5′-GGAAAATGACAAAGAACAGCTCA-3′, a probe 5′-FAM-CAATTTCTACACGAGATCCTCTCTC-BHQ1-3′, and a series of reverse primers 5′-AAATCTTTCTCCTGCTCAGTGAXXXX-3′ for E542K, and 5′-GACTCCATAGAAAATCTTTCTCCTXXXX-3′ for E545K. All primers were selected using the PIK3CA gene (GenBank ID NG_012113.2). XXXX means four nucleotides of the primer 3′-terminus, presented in the text as an abbreviation of the whole primer. The symbol “*” in primer sequences means phosphate group modified with 1,3-dimethylimidazolidine-2-imine moiety (phosphoryl guanidine (PG) modification). PG modification is presented in Figure 1. Boldly marked nucleotides represent mismatched nucleotides concerning the wild-type DNA sequence. Reverse primer 5′-PIK3CA2K-3′ (5′-TGTGACTCCATAGAAAATCTTTC-3′) was used as a reference primer (PIK3CA-ref) to compare the data between various PCR experiments. The ΔCq values were calculated and used for the further analysis of the primers’ efficacy. NTC in all tables means no template control.

### 2.4. Clinical Samples and DNA Extraction

DNA was extracted from FFPE tumor tissue sections using QIAamp DNA FFPE Tissue Kit (Qiagen, Hilden, Germany) following the manufacturer′s protocol. FFPE sections were obtained from 46 breast cancer patients that have been operated on in 14 regional cancer centers across the Siberian and the Far Eastern Federal Districts of Russia. The study was conducted according to the guidelines of the Declaration of Helsinki and was approved by the Local Medical Ethical Committee of ICBFM SB RAS (N7 meeting, 10 August 2022). All patients participating in the study signed informed consent.

### 2.5. Droplet Digital PCR

The ddPCR was performed using the QX200 system (Bio-Rad, Hercules, CA, USA) according to the manufacturer′s recommendations. The reaction mixture in a volume of 20 µL contained 1× ddPCR master mix (Bio-Rad, Hercules, CA, USA), 0.9 µM PIK-1/2 primers, 0.25 µM PIK-WT1/E542K probes for E542K and PIK-WT2/E545K probes, and approximately 10^4^–10^3^ copies of the tested plasmid standard or 3–30 ng of DNA from FFPE samples. The entire reaction mixture with 70 µL of droplet generation oil (Bio-Rad, Hercules, CA, USA) was loaded into a disposable plastic cartridge (Bio-Rad, Hercules, CA, USA) and placed in the droplet generator. After processing, the droplets obtained from each sample were transferred to a 96-well PCR plate (Eppendorf, Hamburg, Germany). The amplification was carried out using T100TM Thermal Cycler (Bio-Rad, Hercules, CA, USA) according to the program: 95 °C for 10 min followed by 45 cycles of PCR amplification (94 °C for 30 s and 57 °C for 60 s), and 98 °C for 10 min, 2 °C/s ramp rate at all steps. After the PCR, the droplets were counted with the QX100 Droplet Reader. The data obtained were analyzed with QuantaSoft software (Bio-Rad, Hercules, CA, USA).

## 3. Results

### 3.1. Design of Allele-Specific Primers with PG-Modification

**Figure 2:**
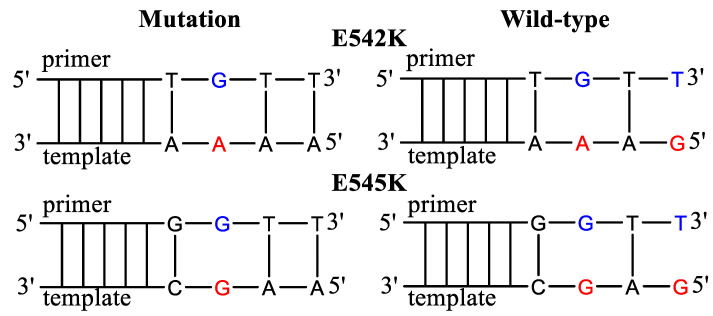
Allele-specific primers for the detection of E542K and E545K mutations. The mismatched nucleotides in the template are marked in red and in the AS primers in blue. The primers harbor additional mismatched nucleotides.

In the present manuscript, we chose AS-PCR for the detection of E542K and E545K mutations in the PIK3CA gene (Figure 2, Table 1). The AS primers were selected, taking into account the possible formation of dimers and an annealing temperature of 62 °C. For each mutation, a set of AS primers was devised with the PG modification of various phosphates and additional mismatches. The AS primers’ 3′-end mismatch concerning WT DNA was G/T (Pyr/Pur) as for the previously investigated in our laboratory case of KRAS G12V mutation [45]. For the G12V mutation, the discrimination efficiency was not satisfying. The mismatch G/T on the 3′-terminus of the elongating chain usually has one of the lowest specificities. Polymerases from different organisms show decreasing discrimination of nucleotide mismatches in the following order: Pur/Pur > Pyr/Pyr > Pur/Pyr = Pyr/Pur [55]. However, increasing the specificity of mutations with such “unfavorable” mismatches is very important for improving clinical outcomes.

**Figure 3:**
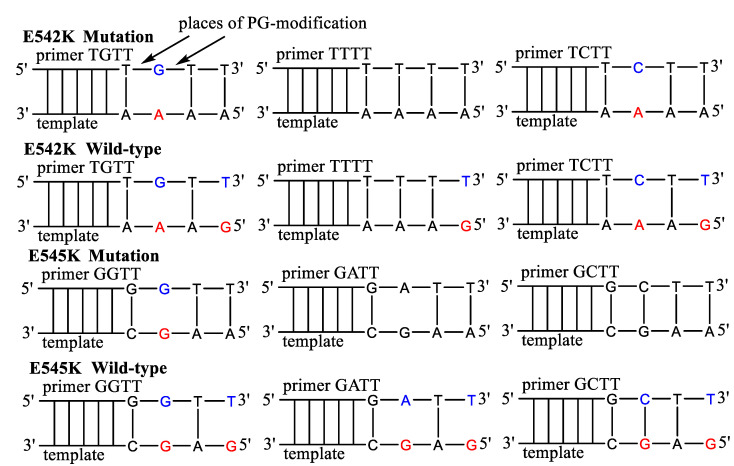
The sequence of PG-modified primers for E542K and E545K mutations detection. The mismatched nucleotides in the template are marked by red, in the primers by blue. The primers harbor additional mismatched nucleotides.

According to previously published data [45], primers with PG-modification at the second or third internucleotide phosphate were chosen. Thus, the influence of the PG groups’ location and the primers’ sequence was investigated using native and PG-modified AS primers (Figure 3, Table 2). The effect of additional mismatches was taken into account by a design of primers with one mismatch at 3′-end or an additional mismatch of AS primers. The AS primers were tested in qPCR using plasmids with PIK3CA gene fragments. Common forward primer and TaqMan probe were used for all AS primers. As a control for each PCR plate, a reference primer PIK3CA-ref, flanking mutations, was used.

### 3.2. PG-Modified Primers Specificity

The specificity of AS primers was investigated using the plasmids with PIK3CA gene fragments of wild-type and bearing mutations. Currently, 1% of mutant DNA on the WT DNA background is acknowledged as a standard sensitivity of AS-PCR for mutation detection. The specificity of AS primers was calculated using the equation ΔCq = Cq_WT_ − Cq_1%_ showing the difference between the reaction with WT DNA and the 1% mutant DNA (total 10^4^ copies per reaction, Table 3). To evaluate the primer design, the PCR efficiency for PG primers has been calculated using a serial dilution of a template (Table 3 and  Table S1). PCR efficiency for non-modified primer T**G**T**T** and G**G**T**T** is slightly higher than 100%, indicating normal reaction conditions and the proper native primers’ design. However, no PG-modified primers demonstrated PCR efficiency higher than 90%. For several primers, the efficacy was lower than 80%, making impossible the detection of low mutation percent. Primers T*GT**T**, TG*T**T**, T*TT**T**, G*AT**T**, and GA*T**T** with PCR efficiencies lower than 80% and the low-specificity primers TT*T**T** and GC*T**T** were excluded from most further PCR experiments.

DNA samples with various mutation percentages were used to determine the specificity of the AS primers. Overall DNA concentration was 10^4^ copies per reaction, and mutation percent was 0.1%, 0.5%, and 1%. However, the 0.1% variant allele fraction (VAF) shows low specificity caused by reaching the limit of qPCR sensitivity (~10 copies per reaction, data not shown). The robust mutation detection was obtained using 0.5% VAF and 10^4^ copies of total DNA per reaction (Table 4). The assay specificity increased with the introduction of a PG modification. Interestingly, the primer G*CT**T** without an additional mismatch showed the best discrimination between WT and mutated DNA. However, the high Cq values were obtained with some AS primers (Table 4), making impossible the detection of low mutated DNA amount.

An additional experiment with a total of 10^5^ DNA copies per reaction was done to evaluate the possibility of detecting a small mutated DNA amount (Table S2). PG-modified primers showed better specificity than native primers. However, all the primers were not able to detect 0.1% of mutated DNA with reliable specificity (see ΔCq Table S2, ΔCq_WT-1%_ ~1). In most cases, the Cq(1%) values for the PG primers were 3–5 cycles higher than for the non-modified primer, which could hinder the detection of a low VAF.

### 3.3. Assay Performance on the Formalin-Fixed Paraffin-Embedded (FFPE) Tissues

**Figure 4:**
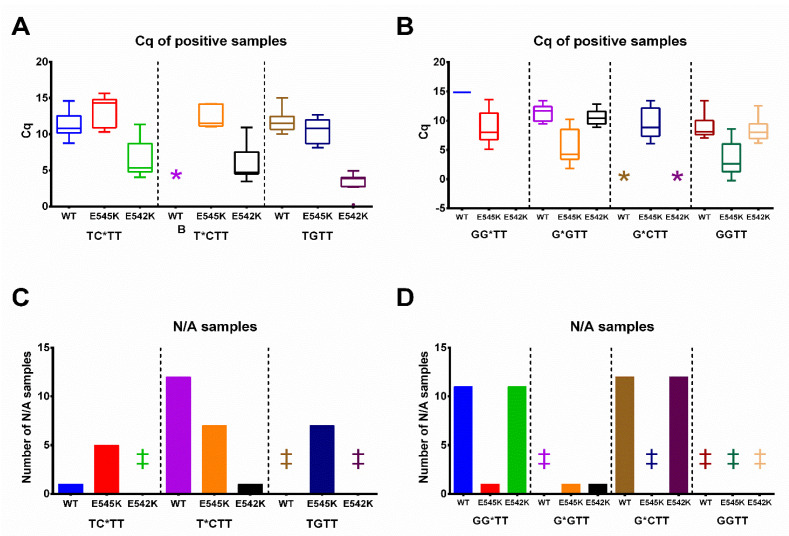
*PIK3CA* E542K (**A**,**C**) and E545K (**B**,**D**) mutation status analysis in FFPE samples. Three types of samples according to dPCR data were used: (1) WT; (2) E542K positive; (3) E545K positive. The clinical samples are coded according to ddPCR mutation status analysis. The mutation percent for the E542K positive samples (left, for TC*T**T**, T*CT**T**, and T**G**T**T** primers) are indicated as 0.56%, 3.72%, 5.76%, 6.21%, 7.03%, 13.53%, 20.91%. The mutation percent for the E542K positive samples (right, for GG*T**T**, G*GT**T**, G*CT**T**, and G**G**T**T** primers) are indicated as 0.56%, 3.72%, 5.76%, 6.21%, 7.03%, 13.53%, 20.91%. (**A**,**B**). A Tukey plot of ΔΔCq values from samples with a positive amplification signal. The box always extends from the 25th to the 75th percentiles, and whiskers represent a 1.5-fold interquartile range. The y-axis marks ΔΔCq values: the difference between ΔCq for 1% positive mutation control and the ΔCq value for each sample. The x-axis represents the type of sample (WT, E542K, E545K-positive) and the AS primer. * marks cases with no positive amplification signal and retrieved Cq values. (**C**,**D**). The number of samples with negative amplification results and no Cq values (N/A). The y-axis marks the number of negative samples; the x-axis represents the type of sample (WT, E542K, E545K-positive) and the AS primer. ‡ marks cases with no negative samples.

Validation of primers was performed using DNA samples from patients with breast cancer. The 46 samples with unknown PIK3CA status were subjected to the analysis. All patients participating in the study signed informed consent. DNA was extracted from FFPE tumor tissue sections using FFPE Tissue Kit (see Experimental part) following the manufacturer′s protocol. The ddPCR analysis was used to validate the data obtained using the proposed qPCR assay in clinical samples (Tables S3 and S4). The reference primer PIK3CA-ref was used instead of AS primers as a control for the total DNA amount in each sample. For all samples, ΔCq(sample) = Cq(AS primer)–Cq(PIK3CA-ref) values were calculated (Figures S1–S3). As a positive control to set up the positive cut-off, a sample with 1% mutated DNA was used (a total of 10^4^ DNA copies per reaction). If ΔCq(sample) < ΔCq(1% reaction), the sample was marked as mutation-positive. The assay results are presented in Figure 4 and Figures S1–S3, which show the PIK3CA E545K and E542K analysis of three types of clinical samples: (1) WT; (2) E542K positive; and (3) E545K positive.

The assay specificity for the PG-modified primers was much higher than for the non-modified G**G**T**T** and T**G**T**T** (Figure 4 and Figures S1–S3). The primers GG*T**T,** G*CT**T,** and T*CT**T** showed good specificity. The Cq values were not retrieved (N/A) for most of the WT and unmatched mutation samples (Figure 4B). The primers G*GT**T,** TC*T**T,** and native T**G**T**T** showed average specificity, while primer G**G**T**T** demonstrated low specificity (Figure 4). The false-positive results were not obtained for all the studied primers (Tables S3 and S4). However, several samples with false-negative results were obtained for each primer. One possible explanation is poor sample quality and a low amount of total DNA. Thus, according to ddPCR data, VAF in all these samples was low, e.g., 0.56%, 0.42%, 0.44%, and 0.05%, close to the detection limit of qPCR. The calculated AS-PCR specificity and sensitivity are presented in Table 5 and Tables S3 and S4.

## 4. Discussion

PG-modification is a non-charged organic residue at the internucleotide phosphodiester fragment [54]. In the previous study [45], the new method AS-PCR method for *KRAS* point mutations detection was proposed. This method used PG-modified primers and provided a good specificity of 0.1% mutation detection in a plasmid model. However, G12V *KRAS* mutation specificity was lower than other mutations related to the unfavorable primer/template 3′-end mismatch (Pyr/Pur). The ability of Taq-polymerase to discriminate mismatches decreases in the following order: Pur/Pur > Pyr/Pyr > Pyr/Pur [55]. Herein, we investigated the same assay for the E542K and E545K mutations in *PIK3CA* gene detection. The low-efficiency AS primers with the same 3′-end mismatch (Pyr/Pur) as for G12V *KRAS* mutation detection were used (Table 2, Figure 3). Considering the data in Table 4, PG primers demonstrated the detection of 0.5% VAF with sufficient specificity. Surprisingly, the primers with a single 3′-end mismatch and PG-modification (T*TT**T** and G*CT**T**) show the same specificity as primers with two mismatches. The primer design with additional mismatches is laborious and requires the examination of several most promising AS primer variants *ad hoc*. Such properties of PG modification may simplify the AS primer’s design. This result was also observed in our previous study [45]. However, the PG mimicking the mismatch is sequence-dependent (*cf.* ΔCq for T*TT**T** and G*CT**T**, Table 4)**.** A limitation of the proposed assay is that PG modification, in some cases, leads to an increase in the Cq value and partly inhibits PCR (Table 3). Despite the lower amplification efficacy compared to the native primers, PG primers demonstrated good specificity. The native primers T**G**T**T** and G**G**T**T** showed low specificity for mutated DNA.

The balance between the primer′s PCR efficiency and specificity is crucial for robust mutation detection. Two options need to be considered during PCR-based test design: (1) high Cq values and better specificity, and (2) relatively low Cq values and medium or low specificity. The first option is applicable to a high amount of mutated DNA. When the amount of target DNA is scarce, low PCR efficacy could result in false-negative results. The second option, low specificity, may lead to a high rate of false-positive results. The exact strategy needs to be chosen *ad hoc*, taking into account the DNA source and the mismatch type. Therefore, the PG primers with low amplification efficacy were excluded from further experiments on the FFPE tissues. FFPE tissues are one of the valuable sources of DNA for clinical screening. Here, FFPE sections were obtained from 46 patients and validated by a ddPCR analysis. PG primers displayed 100% specificity (Table 5), with the highest specificity for the PG-modified primers in the third internucleotide phosphate from the 3′-end (Figure 4). The Cq values were not retrieved (N/A) for most of the WT and unmatched mutation samples (Figure 4B). Some samples, positive by ddPCR, were negative by AS-PCR. The sensitivity of AS-PCR might be affected, considering the use of clinical samples with low DNA amounts. DNA from FFPE samples is fragmented and contains multiple chemical modifications caused by the fixation of FFPE samples. As shown in Figures S1 and S3, several samples have low mutation percentages and cannot be detected by the proposed qPCR assay. The latter is the result of comparison with a highly-sensitive ddPCR assay which allows the detection of mutations unreachable by other diagnostic approaches. The sensitivity of AS-PCR in the range of 0.5–1% is sufficient to detect actionable somatic mutations in DNA samples from FFPE, as these mutations are normally present in a high percentage of tumor cells. In the case of resistant mutations, such as KRAS mutations and anti-EGFR drugs, even a low amount of resistant mutations could not be a basis not to apply the specific therapy [56]. Highly-sensitive methods, such as ddPCR, are preferable for scarce DNA samples, e.g., circulating tumor DNA testing. Therefore, the apparent low sensitivity of PG primers is not a hindrance to actual clinical testing. Removing DNA samples with low mutation percentages led to 100% sensitivity (Table 5, see the sensitivity calculations with and without the low mutation percentages samples).

## 5. Conclusions

In summary, PG-modified primers were used for testing E542K and E545K mutations in the *PIK3CA* gene. Our data showed a possible detection of 50 copies of mutant DNA in a proportion as low as 0.5% of the total DNA with good specificity in the plasmid model system. PG modification in the third internucleotide phosphate mimics an additional mismatch, which may be enough to increase the primer specificity. PG modification may become a universal approach for increasing primer specificity. However, the effect of PG modifications could be template-dependent. The DNA purified from formalin-fixed paraffin-embedded (FFPE) tissues is highly fragmented and chemically modified, bearing various DNA-DNA, DNA-protein cross-links, and mutations. These lesions are known to cause DNA polymerase stalling and reduce amplification efficacy. It has not known whether PG-modified primers are suitable for such DNA analysis. Therefore, the devised assay was tested both on the model plasmid system and the DNA from FFPE. The validation of DNA from FFPE clinical samples showed 100% specificity, highlighting the perspective of PG primers for clinical diagnostics. However, several samples with false-negative results were obtained for each primer. The best sensitivity values were 78% (E545K mutation) and 86% (E542K mutation) for PG-modified primers. The VAF in all false-negative samples was low, e.g., 0.56%, 0.42%, 0.44%, and 0.05%, close to the detection limit of qPCR. The primary AS-qPCR has a detection limit close to 1% VAF. The obtained data suggest that the real sensitivity value provided by PG modification may be higher. Excluding samples, which cannot be analyzed by qPCR, leads to a sensitivity close to 100%. Nevertheless, further studies are needed to evaluate the utility of this test in assessing the clinical significance of PG modification for mutation detection. The PG modification may turn into semi-universal technology for improving primers specificity and selectivity.

## Figures and Tables

**Table 1:** AS-PCR Primers for PIK3CA E542K and E545K mutation detection.

Mutation	Reverse Primers/Template Fragment	Abbreviation
E542K Nucleotide (c.1624G>A) Amino Acid (p.E542K) COSMIC ID (COSV55873227)	5′-AAATCTTTCTCCTGCTCAGTGAT**G**T**T**-3′	T**G**T**T**
	WT DNA 5′-AAATCTTTCTCCTGCTCAGTGATTTC-3′	-
	Mutant DNA 5′-AAATCTTTCTCCTGCTCAGTGATTTT-3′	-
E545K Nucleotide (c.1633G>A) Amino Acid (p.E545K) COSMIC ID (COSV55873239)	5′-GACTCCATAGAAAATCTTTCTCCTG**G**T**T**-3′	G**G**T**T**
	WT DNA 5′-GACTCCATAGAAAATCTTTCTCCTGCTC-3′	-
	Mutant DNA 5′-GACTCCATAGAAAATCTTTCTCCTGCTT-3′	-

Boldly marked nucleotides represent mismatched nucleotides in relation to the WT DNA sequence.

**Table 2:** AS primers for the E542K and E545K mutation detection.

Mutation/Method	Reverse Primers	Abbreviation
E542K/qPCR	5′-AAATCTTTCTCCTGCTCAGTGAT**G**T**T**-3′	T**G**T**T**
	5′-AAATCTTTCTCCTGCTCAGTGATG*T**T**-3′	TG*T**T**
	5′-AAATCTTTCTCCTGCTCAGTGAT*GT**T**-3′	T*GT**T**
	5′-AAATCTTTCTCCTGCTCAGTGATT*T**T**-3′	TT*T**T**
	5′-AAATCTTTCTCCTGCTCAGTGAT*TT**T**-3′	T*TT**T**
	5′-AAATCTTTCTCCTGCTCAGTGATC*T**T**-3′	TC*T**T**
	5′-AAATCTTTCTCCTGCTCAGTGAT*CT**T**-3′	T*CT**T**
E545K/qPCR	5′-GACTCCATAGAAAATCTTTCTCCTG**G**T**T**-3′	G**G**T**T**
	5′-GACTCCATAGAAAATCTTTCTCCTGG*T**T**-3′	GG*T**T**
	5′-GACTCCATAGAAAATCTTTCTCCTG*GT**T**-3′	G*GT**T**
	5′-GACTCCATAGAAAATCTTTCTCCTGA*T**T**-3′	GA*T**T**
	5′-GACTCCATAGAAAATCTTTCTCCTG*AT**T**-3′	G*AT**T**
	5′-GACTCCATAGAAAATCTTTCTCCTGC*T**T**-3′	GC*T**T**
	5′-GACTCCATAGAAAATCTTTCTCCTG*CT**T**-3′	G*CT**T**
ddPCR	5′-AAGAACAGCTCAAAGCAATTTCTA-3′	PIK-1
	5′-TTTAGCACTTACCTGTGACTCCA-3′	PIK-2
	5′-FAM-CGAGATCCTCTCTCTGAAATCAC-BHQ2-3′	PIK-WT1
	5′-HEX-CGAGATCCTCTCTCTAAAATCAC-BHQ2-3′	PIK-E542K
	5′-FAM-AGAAAATCTTTCTCCTGCTCAGT-BHQ2-3′	PIK-WT2
	5′-HEX-AGAAAATCTTTCTCCTGCTTAGT-BHQ2-3′	PIK-E545K

The symbol “*” indicates a PG modification location. Boldly marked nucleotides represent mismatched nucleotides in relation to the WT DNA sequence.

**Table 3:** AS-PCR *PIK3CA* mutation detection using WT DNA (total 10^4^ copies per reaction) and 1% mutant DNA on the background of WT DNA.

Primers		Cq		ΔCq	PCR Efficiency, %
		WT	1%	Cq_WT_ − Cq_1%_	
E542K	T**G**T**T**	34.5 ± 0.5	33.5 ± 0.1	1.0	101.4
	TG*T**T**	N/A	N/A	-	n.d.
	T*GT**T**	37.8 ± 0.2	36.1 ± 0.4	1.7	67.0
	TC*T**T**	39.6 ± 0.4	37.3 ± 0.1	2.3	85.4
	T*CT**T**	37.8 ± 0.3	35.8 ± 0.2	2.0	86.3
	TT*T**T**	35.6 ± 0.3	35.5 ± 0.3	0.1	n.d.
	T*TT**T**	35.8 ± 0.1	34.7 ± 0.2	1.1	75.0
E545K	G**G**T**T**	31.6 ± 0.1	31.2 ± 0.1	0.4	107.2
	GG*T**T**	42.9 ± 0.4	39.0 ± 0.4	3.9	87.6
	G*GT**T**	39.4 ± 0.5	36.9 ± 0.1	2.5	83.6
	GA*T**T**	38.1 ± 0.1	36.5 ± 0.2	1.6	74.7
	G*AT**T**	42.1 ± 0.3	39.3 ± 0.2	2.8	78.6
	GC*T**T**	35.3 ± 0.1	35.1 ± 0.1	0.2	n.d.
	G*CT**T**	42.2 ± 0.4	39.6 ± 0.3	2.6	88.1

No template control (NTC) was undetermined in all the reactions; N/A indicates that no Cq was retrieved for a typical 45-cycle reaction. The symbol “*” indicates the PG modification location. Boldly marked nucleotides represent mismatched nucleotides concerning the WT DNA sequence. PCR efficiency experiments were evaluated using various amounts of DNA (see Table S1). N.d.—not determined. The PCR efficiency experiment for the primers TG*T**T**, TT*T**T**, and GC*T**T** was not done because of the low specificity.

**Table 4:** *PIK3CA* mutations detection by AS-PCR using various mutation percentages (total 10^4^ copies per reaction).

Primers		Cq			ΔCq = Cq_WT_ − Cq_%_	
		WT	0.5%	1%	WT–0.5%	WT–1%
E542K	T**G**T**T**	33.9 ± 0.5	33.2 ± 0.3	32.8 ± 0.2	0.7	1.1
	T*GT**T**	37.3 ± 0.5	36.2 ± 0.2	35.4 ± 0.3	1.1	1.9
	TC*T**T**	39.5 ± 0.6	37.4 ± 0.1	36.9 ± 0.1	2.1	2.6
	T*CT**T**	38.0 ± 0.7	36.2 ± 0.4	36.0 ± 0.2	1.8	2.0
	T*TT**T**	35.7 ± 0.3	34.7 ± 0.2	34.7 ± 0.4	1.0	1.0
E545K	G**G**T**T**	30.9 ± 0.1	30.7 ± 0.1	30.6 ± 0.1	0.2	0.3
	GG*T**T**	41.4 ± 0.6	39.5 ± 0.3	38.4 ± 0.1	1.9	3.0
	G*GT**T**	38.9 ± 0.6	36.5 ± 0.1	35.9 ± 0.1	2.4	3.0
	GA*T**T**	37.6 ± 0.7	36.2 ± 0.1	35.8 ± 0.2	1.4	1.8
	G*AT**T**	41.2 ± 0.6	39.4 ± 0.4	38.7 ± 0.2	1.8	2.5
	G*CT**T**	42.5 ± 0.7	39.5 ± 0.1	38.5 ± 0.1	3.0	4.0

No template control (NTC) was undetermined in all the reactions. The symbol “*” indicates the PG modification location. Boldly-marked nucleotides represent mismatched nucleotides concerning the WT DNA sequence.

**Table 5:** Validation of AS primers on DNA samples from FFPE.

Primer/Mutation	ΔCq Cut-Off	Sensitivity ^1^	Specificity ^2^
G**G**T**T** E545K	2.84	56%/83% ^3^	100%
GG*T**T** E545K	10.55	67%/100%	100%
G*GT**T** E545K	8.00	78%/100%	100%
G*CT**T** E545K	10.64	67%/100%	100%
T**G**T**T** E542K	4.31	86%/100%	100%
TC*T**T** E542K	7.67	86%/100%	100%
T*CT**T** E542K	7.84	86%/100%	100%

^1^ Sensitivity value = true-positive samples/(false-negative + true-positive samples) × 100%; ^2^ Specificity value = true-negative samples/(false-positive + true-negative samples) × 100%; ^3^ Sensitivity recalculation excluding low VAF and low DNA amount samples (lower than 300 DNA copies per µL). For calculations, see Tables S3 and S4.

## Data Availability

The data sets generated and/or analyzed during the current study are available from the corresponding author on reasonable request.

## Supplementary Materials

The following supporting information can be downloaded at: https://www.mdpi.com/article/10.3390/diagnostics13020250/s1, Figure S1: PIK3CA E545K mutation status analysis of FFPE samples for GGTT, GG*TT, G*GTT, and G*CTT primers; Figure S2: The comparison of the reaction of E545K mutation-specific primers with WT and E542K mutation samples; Figure S3: PIK3CA E542K mutation status analysis in FFPE samples for TGTT, TC*TT, and T*CTT primers; Table S1: PCR efficiency calculations; Table S2: PIK3CA mutations detection by AS-PCR using with various mutation percent (total 10^5^ copies per reaction); Table S3: Validation of AS primers for E545K mutation detection on DNA samples from FFPE; Table S4: Validation of AS-primers for E542K mutation detection on DNA samples from FFPE.
