# Supplementary material for: Allele-Specific PCR for PIK3CA Mutation Detection Using Phosphoryl Guanidine Modified Primers
Source: Diagnostics (Basel). 2023 Jan 9;13(2):250. doi: 10.3390/diagnostics13020250 (PMC9858071; doi:10.3390/diagnostics13020250)
Supplement: Supplementary file 1 [file diagnostics-13-00250-s001.zip › diagnostics-1969253-supplementary.pdf]

Allele-Specific PCR for PIK3CA Mutation Detection Using Phosphoryl Guanidine Modified Primers

Alexey S. Chubarov*, Igor P. Oscorbin, Lidiya M. Novikova, Maxim L. Filipenko, Alexander A. Lomzov and Dmitrii V. Pyshnyi

Institute of Chemical Biology and Fundamental Medicine, SB RAS, 8 Lavrentiev Avenue, 630090 Novosibirsk, Russia

* Correspondence: chubarov@niboch.nsc.ru or chubarovalesha@mail.ru

Table S1. PCR efficiency calculations 1

Table S2. *PIK3CA* mutations detection by AS-PCR using with various mutation percent (total 10^5 copies per reaction). 2

Figure S1. *PIK3CA* E545K mutation status analysis of FFPE samples for GGTT, GG*TT, G*GTT, and G*CTT primers..2

Figure S2. The comparison of the reaction of E545K mutation-specific primers with WT and E542K mutation samples. 3

Figure S3. *PIK3CA* E542K mutation status analysis in FFPE samples for TGTT, TC*TT, and T*CTT primers. 3

Table S3. Validation of AS-primers for E545K mutation detection on DNA samples from FFPE.............4

Table S4. Validation of AS-primers for E542K mutation detection on DNA samples from FFPE.............5

Standard curve data were generated for several native and phosphoryl guanidine (PG) modified primers for mutation detection, and the PCR efficiency for each of these primers was calculated using Thermo Fisher Scientific online calculator (Table S1). Standard curves showing the PCR efficiency values for the several primers generated from a dilution series of mutant DNA.

Table S1. PCR efficiency calculations

	Primers	Y-intercept	R^2	PCR efficiency, %
**E542K**	T**GTT**	-3.2884	0.9960	101.4
	T***GTT**	-4.4895	0.9972	67.0
	T**C***TT	-3.7291	0.9995	85.4
	T***CTT**	-3.7019	0.9974	86.3
	T***TTT**	-4.1161	0.9895	75.0
**E545K**	G**GTT**	-3.1600	0.9988	107.2
	G**G***TT	-3.6632	0.9959	87.6
	G***GTT**	-3.7891	0.9994	83.6
	G**A***TT	-4.1275	0.9906	74.7
	G***ATT**	-3.9694	0.9926	78.6
	G*C**TT**	-3.6446	0.9931	88.1

Table S2. *PIK3CA* mutations detection by AS-PCR using with various mutation percent (total 10^5 copies per reaction).

Primers		Cq				ΔCq		
		WT	0.1%	0.5%	1%	$Cq_{WT} - Cq_{0.1\%}$	$Cq_{WT} - Cq_{0.5\%}$	$Cq_{WT} - Cq_{1\%}$
E542K	TGTT	31.1 ± 0.3	31.1 ± 0.2	30.9 ± 0.4	30.3 ± 0.2	0.0	0.2	0.8
	TC*TT	35.7 ± 0.2	35.6 ± 0.2	34.3 ± 0.1	33.3 ± 0.2	0.1	1.4	2.4
	T*CTT	35.4 ± 0.3	34.8 ± 0.2	34.4 ± 0.3	33.6 ± 0.2	0.6	1.0	1.8
E545K	GGTT	28.0 ± 0.3	27.9 ± 0.2	27.8 ± 0.2	27.8 ± 0.1	0.1	0.2	0.2
	GG*TT	39.0 ± 0.2	38.1 ± 0.2	36.3 ± 0.1	35.3 ± 0.1	0.9	2.7	3.7
	G*GTT	36.2 ± 0.2	35.2 ± 0.2	33.2 ± 0.1	33.0 ± 0.3	1.0	3.0	3.2
	G*CTT	39.1 ± 0.2	38.1 ± 0.2	36.6 ± 0.2	36.2 ± 0.3	1.0	2.5	2.9

No template control (NTC) was undetermined in all the reactions, A symbol "*" indicates PG-modification location. Boldly marked nucleotides represent mismatched nucleotides in relation to the WT DNA sequence.

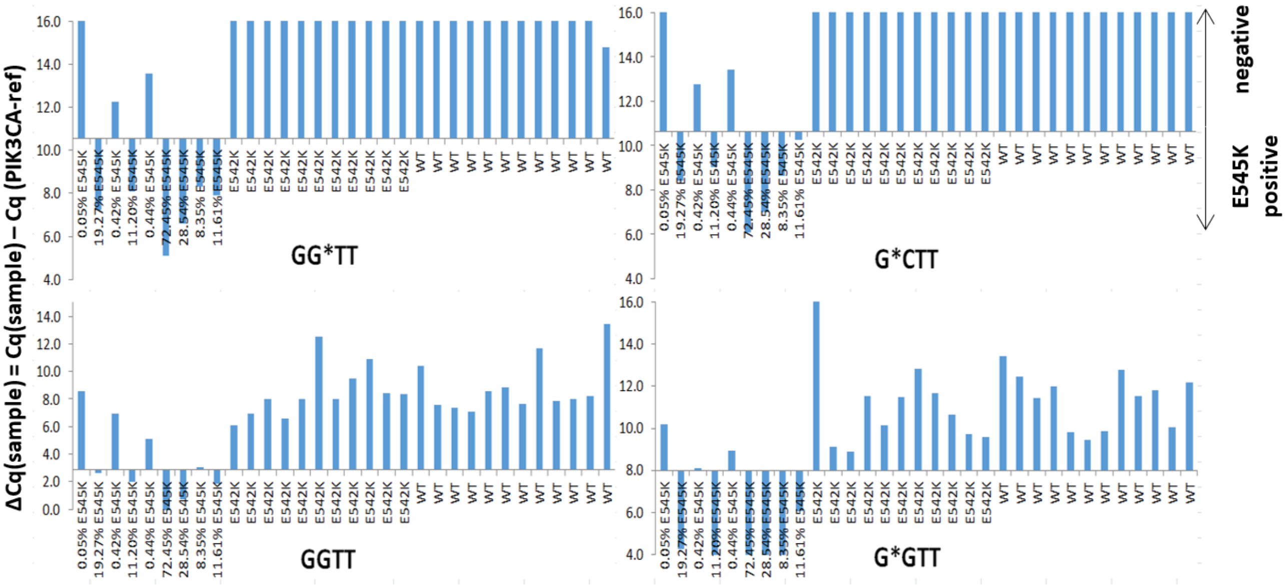

Figure S1. *PIK3CA* E545K mutation status analysis of FFPE samples for GGTT, GG*TT, G*GTT, and G*CTT primers. Three types of samples according to ddPCR data were used: 1) WT; 2) E542K positive; 3) E545K positive. The clinical samples are coded according to ddPCR mutation status analysis. The mutation percent for the E545K positive samples is also indicated (0.05%, 0.42%, 0.44%, 8.35%, 11.20%, 11.61%, 19.27%, 28.54%, 72.45% mutation percent). For each primer the results presented in ΔCq = Cq(sample) - Cq(PIK3CA-ref) values on the y-axis. If ΔCq(sample) < ΔCq(1% mutated DNA of a total of 10^4 DNA copies per reaction for the primers), the sample is marked as positive for the mutation. For the positive samples, the bar is below the x-axis.

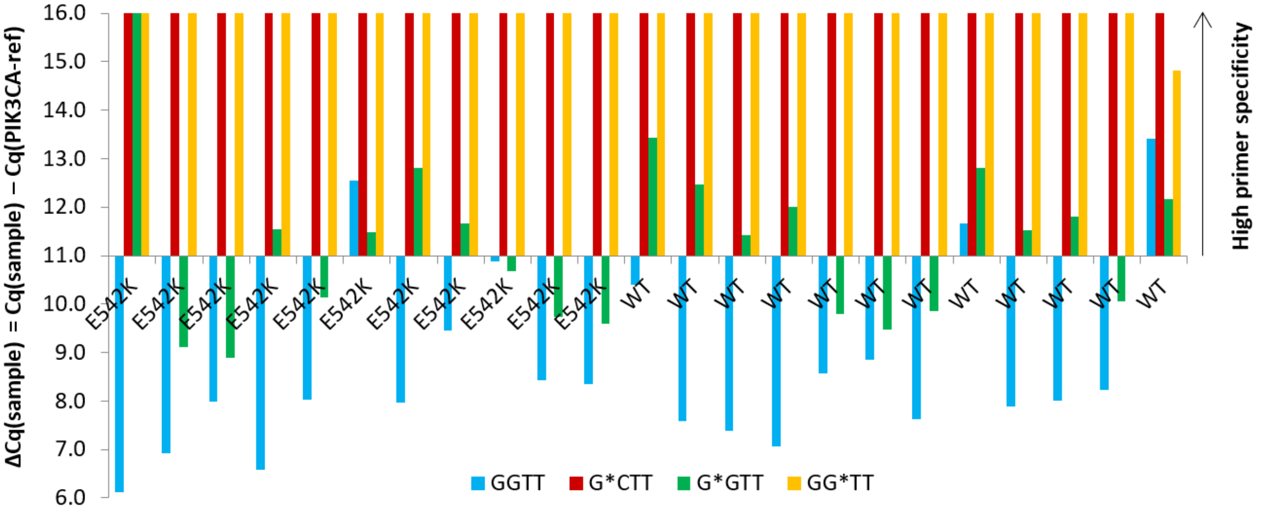

Figure S2. The comparison of the reaction of E545K mutation-specific primers with WT and E542K mutation samples. Primers G**G***TT and G*CT**T** show relatively higher cycles which means that the amplification efficiency of the reaction is low. Primer G***GTT** has average specificity followed by low specific G**GTT**.

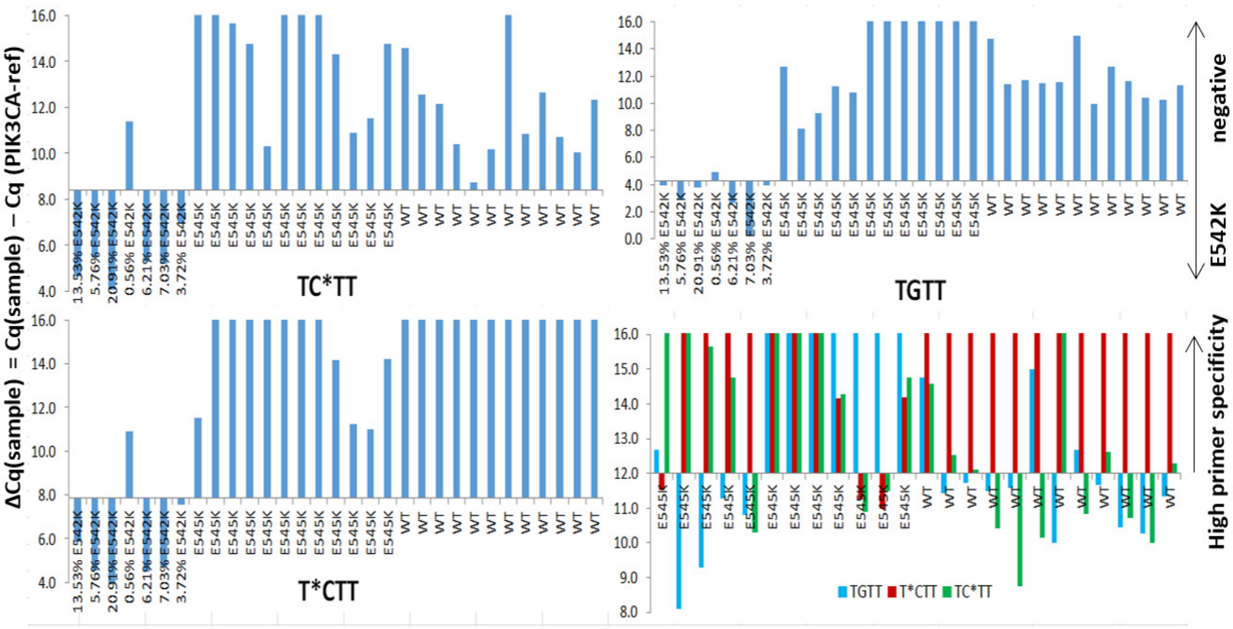

Figure S3. *PIK3CA* E542K mutation status analysis in FFPE samples for T**GTT**, T**C***T**T**, and T***CTT** primers. Three types of samples according to dPCR data were used: 1) WT; 2) E542K positive; 3) E545K positive. The clinical samples are coded according to ddPCR mutation status analysis. The mutation percent for the E542K positive samples is also presented (0.56%, 3.72%, 5.76%, 6.21%, 7.03%, 13.53%, 20.91% mutation percent). For each primer the results presented in ΔCq = Cq(sample) - Cq(PIK3CA-ref) values on the y-axis. If the ΔCq(sample) < ΔCq(1% mutated DNA of a total of 10^4 DNA copies per reaction for the primers), then the sample is marked as positive for the mutation. For the positive samples, the bar is below the x-axis. The comparison of the reaction of E542K mutation-specific primers with WT and E545K mutation samples are presented in the bottom right. Primer T***CTT** shows relatively higher cycles which means that the amplification efficiency of the reaction is low. Primers T**GTT** and T**C***T**T** have average specificity followed.

Table S3. Validation of AS-primers for E545K mutation detection on DNA samples from FFPE.

**Status ddPCR**	**Status AS-PCR**			
	GGTT	GG*TT	G*GTT	G*CTT
0.05% E545K	**WT**	**WT**	**WT**	**WT**
19.27% E545K	E545K	E545K	E545K	E545K
0.42% E545K	**WT**	**WT**	E545K	**WT**
11.20% E545K	E545K	E545K	E545K	E545K
0.44% E545K	**WT**	**WT**	**WT**	**WT**
72.45% E545K	E545K	E545K	E545K	E545K
28.54% E545K	E545K	E545K	E545K	E545K
8.35% E545K	**WT**	E545K	E545K	E545K
11.61% E545K	E545K	E545K	E545K	E545K
E542K	WT	WT	WT	WT
E542K	WT	WT	WT	WT
E542K	WT	WT	WT	WT
E542K	WT	WT	WT	WT
E542K	WT	WT	WT	WT
E542K	WT	WT	WT	WT
E542K	WT	WT	WT	WT
E542K	WT	WT	WT	WT
E542K	WT	WT	WT	WT
E542K	WT	WT	WT	WT
E542K	WT	WT	WT	WT
WT	WT	WT	WT	WT
WT	WT	WT	WT	WT
WT	WT	WT	WT	WT
WT	WT	WT	WT	WT
WT	WT	WT	WT	WT
WT	WT	WT	WT	WT
WT	WT	WT	WT	WT
WT	WT	WT	WT	WT
WT	WT	WT	WT	WT
WT	WT	WT	WT	WT
WT	WT	WT	WT	WT
WT	WT	WT	WT	WT
**True positive**	5	6	7	6
**True negative**	23	23	23	23
**False positive**	0	0	0	0
**False negative**	4	3	2	3
**Sensitivity, %**	56 (5/9: 26.6-81.2)	67 (6/9: 35.1-88.3)	78 (7/9: 44.3-94.7)	67 (6/9: 35.1-88.3)
**Sensitivity without 0.05%, 0.42%, 0.45% samples, %**	83 (5/6: 41.8-98.9)	100 (6/6: 64.3-100)	100 (6/6: 64.3-100)	100 (6/6: 64.3-100)
**Specificity, %**	100 (23/23: 87.5-100)	100 (23/23: 87.5-100)	100 (23/23: 87.5-100)	100 (23/23: 87.5-100)

Sensitivity value = true-positive samples / (false-negative + true-positive samples) × 100%
Specificity value = true-negative samples / (false-positive + true-negative samples) × 100%
Confidence interval (Wald interval) was calculating using online calculator https://measuringu.com/calculators/wald/#wilson

Table S4. Validation of AS-primers for E542K mutation detection on DNA samples from FFPE.

Status ddPCR	Status AS-PCR		
	T**G**TT	T**C***TT	T***C**TT
13.53% E542K	E542K	E542K	E542K
5.76% E542K	E542K	E542K	E542K
20.91% E542K	E542K	E542K	E542K
0.56% E542K	**WT**	**WT**	**WT**
6.21% E542K	E542K	E542K	E542K
7.03% E542K	E542K	E542K	E542K
3.72% E542K	E542K	E542K	E542K
E545K	WT	WT	WT
E545K	WT	WT	WT
E545K	WT	WT	WT
E545K	WT	WT	WT
E545K	WT	WT	WT
E545K	WT	WT	WT
E545K	WT	WT	WT
E545K	WT	WT	WT
E545K	WT	WT	WT
E545K	WT	WT	WT
E545K	WT	WT	WT
E545K	WT	WT	WT
WT	WT	WT	WT
WT	WT	WT	WT
WT	WT	WT	WT
WT	WT	WT	WT
WT	WT	WT	WT
WT	WT	WT	WT
WT	WT	WT	WT
WT	WT	WT	WT
WT	WT	WT	WT
WT	WT	WT	WT
WT	WT	WT	WT
WT	WT	WT	WT
**True positive**	6	6	6
**True negative**	24	24	24
**False positive**	0	0	0
**False negative**	1	1	1
**Sensitivity, %**	86<br>(6/7: 46.7-99.5)	86<br>(6/7: 46.7-99.5)	86<br>(6/7: 46.7-99.5)
**Sensitivity without 0.56% sample, %**	100<br>(6/6: 64.3-100)	100<br>(6/6: 64.3-100)	100<br>(6/6: 64.3-100)
**Specificity, %**	100<br>(24/24: 88.0-100)	100<br>(24/24: 88.0-100)	100<br>(24/24: 88.0-100)

Sensitivity value = true-positive samples / (false-negative + true-positive samples) × 100%
Specificity value = true-negative samples / (false-positive + true-negative samples) × 100%
Confidence interval (Wald interval) was calculating using online calculator https://measuringu.com/calculators/wald/#wilson
